# Modeling and Simulation of a Novel Relay Node Based Secure Routing Protocol Using Multiple Mobile Sink for Wireless Sensor Networks

**DOI:** 10.1155/2015/495945

**Published:** 2015-10-01

**Authors:** Madhumathy Perumal, Sivakumar Dhandapani

**Affiliations:** ^1^Anna University, Chennai 600025, India; ^2^ECE Department, Easwari Engineering College, Chennai 600089, India

## Abstract

Data gathering and optimal path selection for wireless sensor networks (WSN) using existing protocols result in collision. Increase in collision further increases the possibility of packet drop. Thus there is a necessity to eliminate collision during data aggregation. Increasing the efficiency is the need of the hour with maximum security. This paper is an effort to come up with a reliable and energy efficient WSN routing and secure protocol with minimum delay. This technique is named as relay node based secure routing protocol for multiple mobile sink (RSRPMS). This protocol finds the rendezvous point for optimal transmission of data using a “splitting tree” technique in tree-shaped network topology and then to determine all the subsequent positions of a sink the “Biased Random Walk” model is used. In case of an event, the sink gathers the data from all sources, when they are in the sensing range of rendezvous point. Otherwise relay node is selected from its neighbor to transfer packets from rendezvous point to sink. A symmetric key cryptography is used for secure transmission. The proposed relay node based secure routing protocol for multiple mobile sink (RSRPMS) is experimented and simulation results are compared with Intelligent Agent-Based Routing (IAR) protocol to prove that there is increase in the network lifetime compared with other routing protocols.

## 1. Introduction

Wireless sensor network is self-organized, scattered, sensing, and data propagation network. Nodes are resource constrained tiny autonomous devices. They are used to sense the environmental conditions in their immediate surroundings and transmit the data to the sink [1]. WSN can be used to monitor environment and collect data from massive fields at a very low cost and with less manpower. Mobile devices are attached to the sensors with robotic elements and the data is exchanged between sensors nodes to drive applications like traffic monitoring, environmental monitoring, habitat monitoring, and climate study [2, 3].

*Data Gathering Using Mobile Sink.* Data gathering using mobile sink minimizes the precious sensor node energy. The effectiveness of it can be determined by the total sensor energy saved and the time saved in gathering the sensory data from its field or from the trajectory length of the mobile sinks [4]. Mobile sink in WSN optimizes the energy consumption and reduces the delay observed during data gathering [5, 6].

*Routing and Issues on Mobile Sink Data Gathering.* The sink possesses significant replenishable energy resource; it should move closer to the subset of sensor devices to collect all the recorded data. The energy consumption during this process is very minimal. The sink should be within a sensor range for single hop communication and remain within the transmitter range for successful communication. This problem becomes severe when the number of sensors is increased in a particular area. This results in inadequacy in network communication time to upload the data; the node has to wait till the sink returns back. As a result, high delivery delay occurs [7]. Mobile sink is a new challenge for densely deployed nodes and large WSN [8].

*Protocol for Secure Communication in Mobile Sinks.* Securing wireless sensor networks [WSN] is the primary and a daunting task because of its characteristics such as unreliable wireless communication, constraints with respect to the resources, unknown topology, and unprotected environment. The primary security goals here would be to maintain data confidentiality by not leaking data to neighboring node, to provide data freshness, and to provide high availability and authenticity by verifying that the data is always sent by an authentic source.

## 2. Related Literature

Kim et al. [9] proposed Intelligent Agent-Based Routing (IAR) protocol for providing data delivery efficiently to the mobile sink. The IAR protocol is evaluated using mathematical analysis. Results proved that the protocol supports mobility of sink with less overhead and an improvement over the triangular routing problem.

Similarly, [10] have highlighted the difference between a mobile relay and the mobile sink. A mobile relay collects data whenever it is closer to sensor nodes. Mechanical movement is employed to transport the data to the sink. As it does not use the wireless links for transmission to the sink, the latency in delivering data is significant. In contrast, mobile sink performs various operations like distributing the load among the sensor nodes, collecting data continuously from the nodes, and moving slowly and discontinuously in data gathering process.

In one attempt, the mobile sink is a moving strategy based on remaining energy of the sensor nodes which balance the network workload and increase the network life time [11]. Consequently, [12] proposed a query-based data collection scheme in which mobile sink issues queries in the sensing field and the response is received through multihop communication. The problem with such query-based systems is that the sink mobility causes the query and response packets to take different routes. Query-based data collection scheme (QBDCS) consumes lesser energy and delivered packet with minimum latency. Moreover, QBDCS selects the optimal time for sending its query packet and tailored the routing process for sensor nodes for forwarding packets. The performance of the QBDCS was evaluated by comparing with a “Naive” scheme using the OMNeT++ simulation tool.

Reference [13], proposes RECPE a collection protocol which is reliable in a significant high scale WSN for aggregating packets from the source to sink. This protocol has successfully covered all the routes in the network by employing expected transmission count for forward links (ETF) method for constructing a collection tree, thereby reducing the consequence of the asymmetric link in the entire network. Moreover, the proposed protocol also utilized Trickle algorithm and pipeline mechanism. This mechanism minimizes control information and increases the effectiveness of data delivery. Reference [14] proposed a Mobile Sink Based Reliable and Energy Efficient Data Gathering (MSREEDG) technique for gathering data in tree-shaped network topology in WSN and compared with constant pause time method.

Reference [15] uses an agent-based architecture that saves the bandwidth and segregates SAPID (self-organized agent-based architecture for power-aware intrusion detection) in two phases. This agent architecture principally uses a Kohonen Self-Organizing Map to recognize the patterns and identify anomalies appropriately for unauthorized users. Reference [16] tackles the hotspot problem and presents a mobile sink based Routing Protocol (MSRP) for extending network lifetime for cluster based architecture in WSN. MSRP moves to gather the data, which in turn reduces the hotspot problem. Similarly various protocols are discussed in [17–19].

An alternative protocol to minimize the energy consumption and overhead during the data retrieval process is proposed in [20]. Adaptive mobile strategy is implemented for hierarchical networks in a large scale that are presented in [21]. The trajectory of mobile sink is planned in such a way that no multihop relays are required for transmission of data to the sink. Reference [22] presents using multihop forwarding forming a cluster around the expected position of a mobile sink; guaranteeing minimum energy consumption with packet delay is discussed in [23].

A new data gathering scheme named as Maximum Amount Shortest Path to increase the network throughput and to save energy by efficiently assigning the sensor nodes is discussed in [24]. A weighted rendezvous planning (WRP) heuristic is presented in [25]; in this, each sensor node is assigned with a weight appropriate to its hop distance and the number of data packets it forwards. In another attempt [26], mobility patterns for data collection of the sink are presented. Frequent location updates from mobile sinks increase the energy consumption in wireless transmissions [27].

In [28], a model for investigating the joint sink mobility is presented and routing problem is constrained by the sink with a predefined number of locations. The routing protocol between multiple source and sink is proposed in [29]. Reference [30] proposed secure energy efficient data collection for mobile sink using a session based symmetric key cryptography method to increase the life time of the network. Among all other security goals, only authentication of the node and data fairness is considered in this paper, several internal attacks are out of the scope of this paper.

Based on the literature review of the mobile sinks, this paper proposes a protocol called relay node based secure routing protocol for multiple mobile sink, which is used for data gathering, and the simulation results are compared with IAR protocol. The results are demonstrated in such a way that the proposed protocol performs better in terms of performance metrics like delivery ratio and energy efficiency and reduces the delay and overhead in the WSN.

The reminder of the paper is prepared in such a way that Section 3 discusses the problem formulation, architecture, data gathering routing protocol, relay node selection algorithm, and security protocol in detail.  Section 4 discusses the experimentation and simulation results in detail using the proposed method and also gives a detailed comparison of the results to show the superiority of the proposed method. Then the paper discusses the results and finally concludes.

## 3. The Proposed Solution

### 3.1. Problem Formulation

Reference [14] proposed MSREEDG protocol for gathering data. The sink's next moving position is determined by using a Biased Random Walk model. The optimal path for data transmission is estimated by rendezvous point selection method and splitting tree process. When the sensor senses the data and when it is ready for transmission, the data are encoded and transmitted to the sink, and then it decodes the data and the resulting message is stored in local buffer. After decoding all the blocks, the original data bundle is reconstructed by the mobile sink. The increase in the packet loss is prevented by maximizing the sink pause time. In this process only single sink is used and when sink is out of its range the source has to wait for the sink till it comes back to its region.

This process can be enhanced for multiple numbers of sinks and designing an efficient routing protocol for data gathering. Here a relay node based secure routing protocol using multiple mobile sink for data gathering for WSN is proposed.

### 3.2. Proposed Architecture

**Figure 1:**
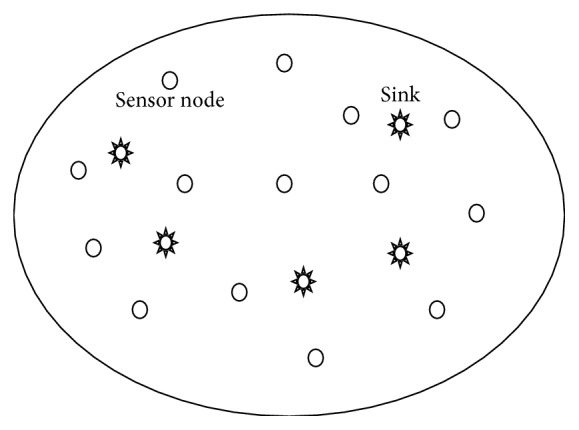
Proposed architecture of multiple mobile sink.

The source and sink know their location. It is also assumed that multiple sinks move in the network. The proposed architecture is shown in Figure 1.

### 3.3. Data Gathering Routing Protocol

 Let *S* be the source node.
 Let *D* be the sink node.
 Let QP be query packet.
 Let ReP_mr_ be rendezvous point.
 Let RN_*i*_ be the relay node.
 Let RP_*n*_ be the new relay path.
 Let RP_*o*_ be the old relay path.
 Let RP_seq be the sequence number of relay path.
 Let RP_mes and R_CLR be the relay path setup and clear message with RP_seq.

(1) If event occurs, initially, a rendezvous point (ReP_mr_) is selected.
(2) Then sink has to transmit QP to ReP_mr_ once the event occurs.
 The fields in the QP are represented as the following:
   Sink ID.
   Hop count.
   Transmitter distance.
(3) ReP_mr_ broadcasts the QP with hop count counter value as zero.
(4) When the neighboring nodes receive the QP, they rebroadcast the packet by incrementing hop count counter by 1. Thus, as this query propagated, new hop node towards ReP_mr_ is estimated that is in one hop communication distance.
(5) If next new hop node > 1,
   Then
     Two nodes compare the packets arrival time (Tpa)
     The new next hop node with earlier Tpa is chosen
   End if
     Source transmits the path request message (P_REQ) to all its neighbors and the neighbor, node that sends the (P_REP) is chosen as next new hop node.
     If *D* move in its radio range of ReP_mr_
   Then
     
*D* receives the collected data from the ReP_mr_.
     Else
     
*D* chooses RN_*i*_ from its neighbor nodes to transmit the data from ReP_mr_ to sink. (Relay node selection)
   End if
   Here, the node, which is nearest to *D* is selected as RN.
(6) If an event occurs, Ni enclosing it collectively processes the signal. One among the nodes becomes *S* to generate the data reports.
(7) When *S* matches the data sent by QP, the data is forwarded to one hop distance node.
(8) If the next new hop node is failed or its battery is exhausted
   Then
    (1) S→P_REQNeighi,Neighi→P_REPS.
   
*S* chooses the respective Neigh_*i*_ as next new hop node.
   End if.

### 3.4. Relay Node Selection

When data packets are not received by *D* for predefined time interval *T*, it suspects that they are out of the coverage radio range. In order to prevent this action, the following steps are executed.

**Figure 2:**
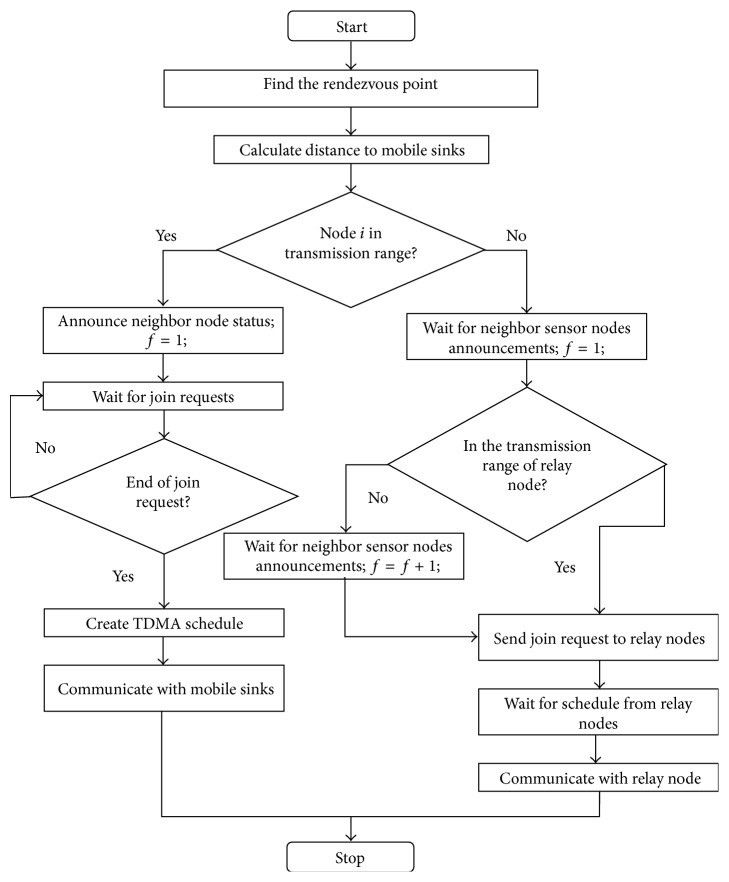
Flowchart of the proposed routing protocol.

 
  (i) ReP_mr_ transmits at least one packet at interval of *T*/*n* period (*n* is integer).
  (ii) If ReP_mr_ does not have data to send in *T*/*n* duration, it transmits NULL packet. Thus, when *S* does not receive the data packet for *T*, it performs the following actions to select the relay node.
(1) Relay node request message (R_REQ) is sent to its Neigh_*i*_:
  (2) $$\begin{array}{c} D\overset{\text{R\_REQ}}{\to }{\text{Neigh}}_{i}. \end{array}$$
(2) Neigh_*i*_ node will reply to the sink:
  (3) $$\begin{array}{c} {\text{Neigh}}_{i}\overset{\text{R\_REP}}{\to }D. \end{array}$$
(3) Sink chooses the node which is nearer to it as immediate relay node.
(4) The relay path message is sent through IRN:
  (4) $$\begin{array}{c} D\overset{\text{RP\_mes}}{\to }{\text{IRN}}_{i}\overset{\text{RP\_mes}}{\to }{ReP}_{mr}. \end{array}$$
(5) When ReP_mr_ receives the RP_mes, data packets are transmitted in the path traversed by RP_mes. This relay path is flagged with RP_seq.
 The following is noted: When *D* moves away from radio range or after completing its relay path setup, there may be possibility of packet drop. This is prevented by ReP_mr_ by caching the overhead packets which are transmitted to *D*. The cached packets are routed to *D* when ReP_mr_ receives RP_mes.
(6) If *D* is again away from its transmission range of IRN_*i*_, then it selects new IRN_*i*_ (as per step (3)).
(7) *D* then transmits RP_mes to ReP_mr_ through the newly selected IRN_*i*_ in separate relay path RP_*n*_ which is flagged with RP_*n*__SEQ.
  (a) When ReP_mr_ receives relay path setup message,
  If RP_*o*_ exists for the same sink
     Then
      (5) $$\begin{array}{c} {ReP}_{mr}\overset{\text{R\_CLR}}{\to }{\text{RP}}_{o}. \end{array}$$
     End if
  When old relay path exists, then ReP_mr_ send R_CLR message along RP_*o*_ which is flagged with RP_*o*__SEQ.
  (b) When the relay path gets a new RP_mes, then it does not remove the RP_*o*_ state. RP_*o*_ state is maintained until the path receives R_CLR for RP_*o*_.  Figure 2 shows the flow chart of the proposed protocol.

### 3.5. Security Protocol for Data Gathering

**Figure 3:**
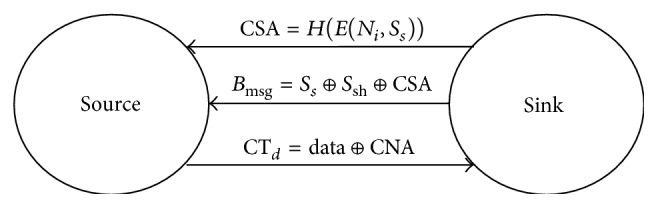
Source and sink communication.

Security protocol securely gathers the sensed data and transmits it to the sink. In this proposed model reactive type of data collection is used. This protocol provides high degree of security with minimum overhead. Symmetric key is used in WSN because of less computation as it uses same keys for encryption and decryption. The source and sink communication is showed in Figure 3.

 Secret key of the sensor node is *S*_*i*_.
 Secret key of the sink node is *S*_*s*_.
 Shared key is *S*_sh_.
 Prime number is *N*_*i*_.
 Code for sink authentication is CSA.
 Code for node authentication is CNA.
 Code for message authentication is CMA.
 Cipher text data is CT_*d*_.
 Hash function is *H*().

#### 3.5.1. The Process at the Sink Node

Consider the following equations:

 CSA = *H*(*E*(*N*_*i*_, *S*_*s*_)).
 Data = CT_*d*_ ⊕ CSA ⊕ *S*_*i*_ = CT_*d*_ ⊕ CNA.

At the sink node, the cipher text is decrypted to get the original message:

(1) When the sink comes into a range of rendezvous point it generates a large prime number.
(2) Sink calculates CSA (CSA = *H*(*E*(*N*_*i*_, *S*_*s*_))).
(3) CSA is broadcasted among the neighbor nodes.
(4) *B*_msg_ is broadcasted to all the nodes (*B*_msg_ = *S*_sh_ ⊕ *S*_*s*_ ⊕ CSA).
(5) Cipher message is received and decrypted with its secret key (Data = CT_*d*_ ⊕ CSA ⊕ *S*_*i*_ = CT_*d*_ ⊕ CNA).
(6) Compare the decrypted data with CMA; if data is same, accept it; else reject the data.
(7) If the data is rejected, NAC is sent to the respective node.
(8) The process is repeated till all the data is transmitted within its time slot.

#### 3.5.2. The Process at Source Node 

Consider the following equations:

 CNA = CSA ⊕ *S*_*i*_.
 CT_*d*_ = Data ⊕ CNA.

At the source node the plain text is encrypted with the secret key.

(1) Sensor node calculates its secret key when CSA is received (CNA = CSA  ⊕  *S*_*i*_).
(2) Sensor receives the *B*_msg_ and calculates *B*_msg_  ⊕  CSA  ⊕  *S*_sh_.
(3) The corresponding result is compared with the sink secret key; if it is same accept the message; else reject the message.
(4) Sensor encrypts its data with secret key and generates its cipher text CT_*d*_ = Data ⊕ CNA.
(5) CMA is added at the end of the packet for verification.
(6) The process is done till the end of the data packets.

## 4. Experimentation and Simulation

NS2 simulation is employed to evaluate relay node based secure routing protocol for multiple mobile sink (RSRPMS). In this case randomly deployed sensor nodes covering the area of 600 × 600 m are varied from 50, 100, 150, and 200, to 250 kbps data rate and nodes are varied from 20 to 100 nodes. The time taken for simulation is 50 sec.

### 4.1. The Performance Evaluation in Terms of Number of Sinks

This section describes the simulation results when sinks are increased as 1, 2, and 5. All the performance parameters were evaluated for node as well as rate. From the simulation result the proposed protocol proves that it can work better when the number of sinks increases. This increases the availability of the network.

### 4.2. Simulation Results for Varying Nodes

#### 4.2.1. Nodes versus Delay

**Figure 4:**
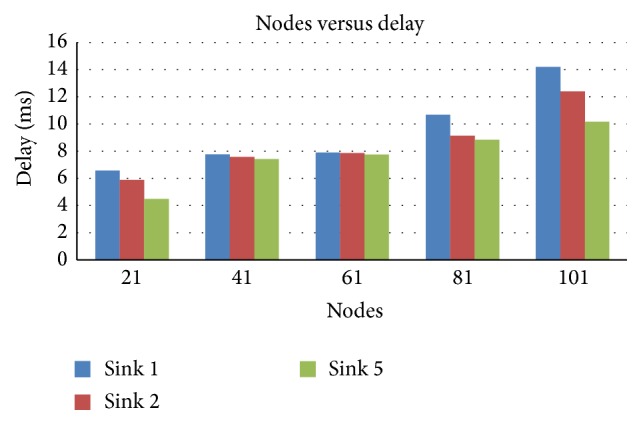
Nodes versus delay in terms of sink.

From Figure 4 the delay keeps reducing when the number of sinks increases. The operation with 5 sinks provides better performance than with lesser number of sinks.

#### 4.2.2. Nodes versus Drop

**Figure 5:**
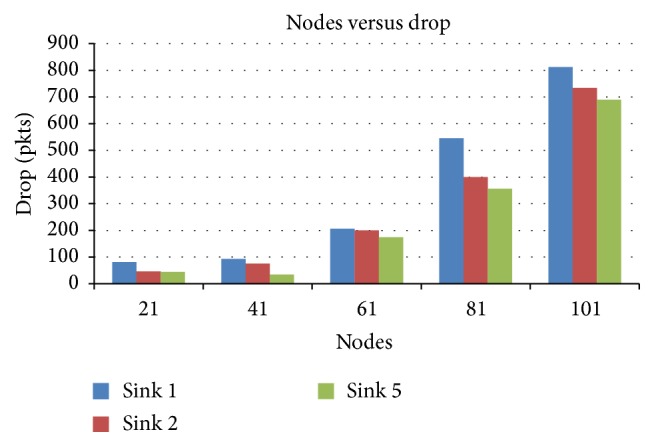
Nodes versus drop in terms of sink.

Figure 5 presents the packet drop for various node scenarios when the number of sinks is varied as 1, 2, and 5. It can be seen that when number of nodes is increased, the drop decreases drastically for 5-sink scenario compared lesser number of sinks.

#### 4.2.3. Nodes versus the Energy

**Figure 6:**
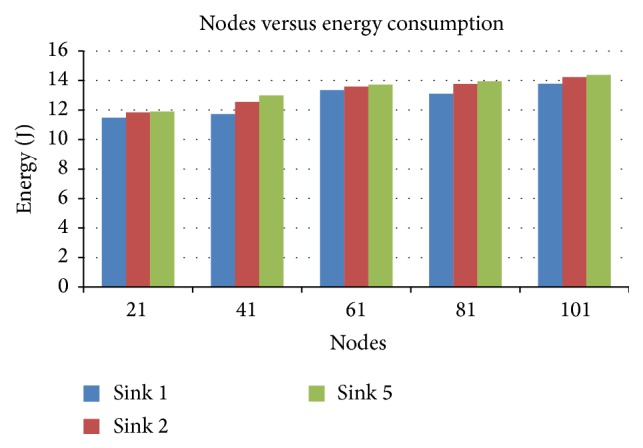
Nodes versus energy in terms of sink.

Figure 6 presents the energy consumed by various nodes when the sink number is varied as 1, 2, and 5. It can be observed that when node number is increased, energy consumption for sink 5 is higher than 1 or 2 sinks.

#### 4.2.4. Nodes versus Overhead

**Figure 7:**
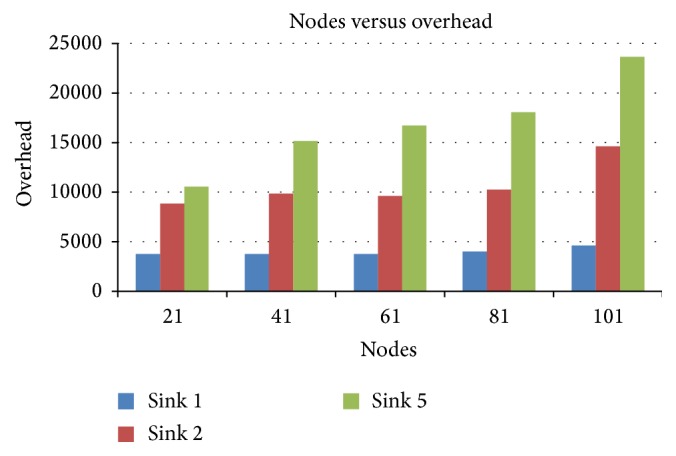
Nodes versus overhead in terms of sink.

Figure 7 presents the overhead for various node scenarios when sinks are varied from 1, 2, and 5. However, the overhead is very low for 1-sink operation compared with 2- or 5-sink operations.

### 4.3. Simulation Results for Varying Data Rate

#### 4.3.1. Rate versus Delay

**Figure 8:**
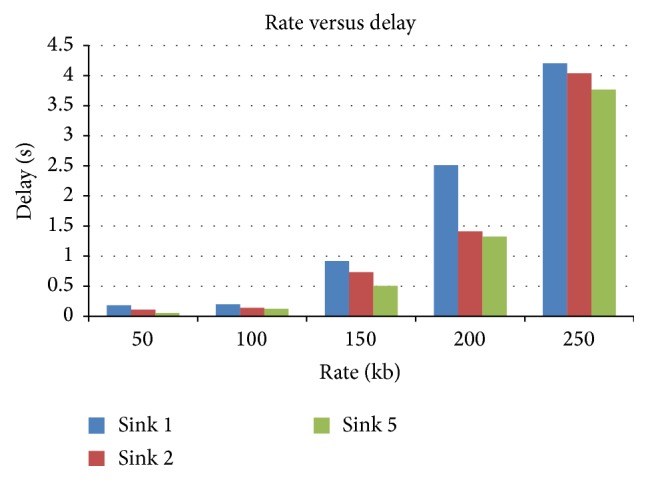
Rate versus delay in terms of sink.

Figure 8 presents the delay versus rate for various rate scenarios when the number of sinks is varied as 1, 2 and 5. It is very clear the delay is minimum for the 5-sink operation and maximum for a single sink operation.

#### 4.3.2. Rate versus Drop

**Figure 9:**
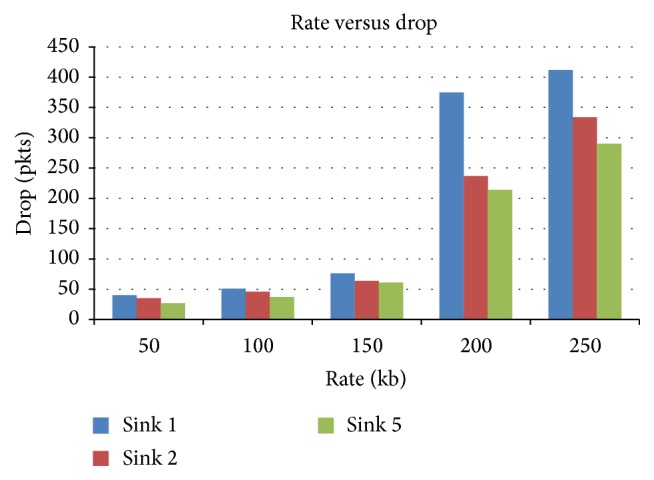
Data rate versus drop in terms of sink.

Figure 9 presents the packet drop for various rate scenarios when sink is varied as 1, 2, and 5. When data rate is increased, the drop increases drastically for 1-sink scenario compared to 2 or more number of sinks.

#### 4.3.3. Rate versus Energy

**Figure 10:**
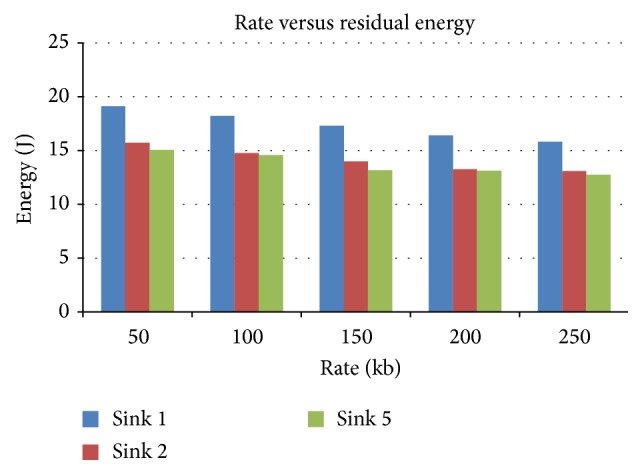
Rate versus energy in terms of sink.

Figure 10 presents the packet energy for various rate scenarios when the sink is varied as 1, 2, and 5; as the rate is increased, energy consumption increases. The residual energy of sink 5 is lesser than sink 1 and sink 2.

#### 4.3.4. Rate versus Overhead

**Figure 11:**
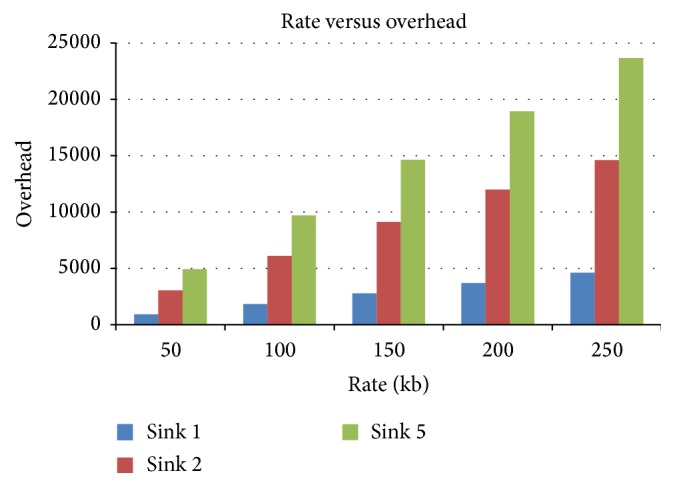
Rate versus overhead in terms of sink.

Figure 11 presents the overhead in terms of data rate for various numbers of sinks. It is observed that overhead is maximum for 5-sink scenario when compared to other sink scenarios.

Table 1 presents the comparison between the RSRPMS and IAR techniques. In terms of delivery ratio under different number of nodes operation, RSRPMS shows better performance than IAR approach by 27%. The packet delivery ratio of RSRPMS is 9.8% higher than IAR approach under different number of rate operations.

**Figure 12:**
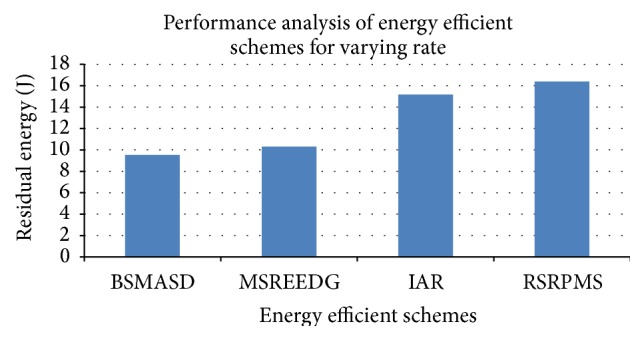
Performance analysis of energy efficient schemes for varying data rate.

Figure 12 shows energy schemes for 200 kbps data rate with 100 nodes. Residual energy of MSREEDG is higher than BSMASD but lesser than RSRPM. RSRPMS is higher than Intelligent Agent-Based Routing protocol by 8.2%. Thus the proposed protocol increases the energy efficiency and reliability of the data. The overall result proves that the proposed protocol increases the network life time with high reliability, integrity, availability with minimum delay, energy, drop, and overhead.

## 5. Conclusion

This paper proposes a reliable and energy efficient WSN routing protocol with minimum delay called relay node based secure routing protocol for multiple mobile sink (RSRPMS). The experimental simulations using the proposed protocol are carried out rigorously and the results are compared with Intelligent Agent-Based Routing (IAR) protocol to prove the superiority in delivery of transmitted data and increase in the network lifetime compared with other routing protocols. The proposed routing protocol performance was also additionally compared with traditional schemes like BSMASD and MSREEDG techniques. The parameters of comparison included packet drop, energy, delay, and overhead. The simulations were carried out using NS2 simulator under various conditions of operations like varying the nodes and data rate. The simulation result proves that the proposed relay node based secure routing protocol used for mobile sink increases the delivery ratio and energy efficiency. Thus the proposed routing protocol increases the network lifetime.

## Figures and Tables

**Table 1:** Delivery ratio of the data gathering schemes.

Data gathering schemes	Delivery ratio (%)	
	Node	Rate
	Nodes: 80	Rate: 200 kbps
BSMASD	0.37426	0.13937
MSREEDG	0.60471	0.31161
IAR	0.55215	0.91863
RSRPMS	0.78017	0.99556
